# Supplementary material for: Endometriosis treatment pathways in the largest private health insurance in Brazil: A real-world data study
Source: PLoS One. 2026 Apr 10;21(4):e0345399. doi: 10.1371/journal.pone.0345399 (PMC13068217; doi:10.1371/journal.pone.0345399)
Supplement: Attachment — Submitted filename: Point.pdf [file pone.0345399.s001.pdf]

Point-by-point response

We sincerely thank the reviewers for their careful evaluation of our manuscript and for the constructive comments provided. We are pleased that Reviewer #1 recognized the relevance and value of our data in highlighting the societal and economic burden of deep endometriosis. We have carefully addressed all suggestions from Reviewer #2, which substantially improved the clarity, clinical accuracy, and scientific rigor of the manuscript.

Regarding the Introduction, we revised the clinical framing of deep endometriosis to prioritize organ infiltration, removing the initial emphasis on the rare involvement of lymph nodes and placing greater focus on bowel, bladder, and ureteral disease. Key symptoms were updated to reflect standard clinical terminology, including dysmenorrhea, deep dyspareunia, dysuria, and dyschezia. Statements concerning surgical management were refined to clarify that surgery is indicated in symptomatic patients refractory to medical treatment, in cases of organ involvement, or in selected patients with infertility, while also emphasizing that surgery does not eliminate the underlying disease process and that recurrence remains possible. Minor semantic adjustments were made to improve accuracy, including explicit reference to treatment timelines.

We also expanded the Introduction to better highlight current challenges and developments in the diagnosis of endometriosis, particularly deep endometriosis. A new paragraph was added addressing limitations in access to specialized imaging, variability in expertise, and the absence of universally adopted classification systems, thereby contextualizing diagnostic delays within contemporary clinical practice.

In the Methods section, we clarified that no standardized classification system (such as rASRM or ENZIAN) was systematically applied, as surgical findings were derived from routine real-world clinical documentation within an administrative database. This clarification directly addresses the reviewer's question regarding classification.

Within the Discussion, we strengthened the interpretation of our findings by explicitly addressing the diagnostic gap in deep endometriosis. We incorporated a sentence linking the delayed surgical treatment observed in patients with deep disease to nonspecific symptom presentation and limited access to specialized imaging. Furthermore, following the paragraph describing MRI performance, we added a dedicated discussion on the role of non-invasive diagnostic tools, emphasizing their importance in reducing diagnostic delays, avoiding unnecessary invasive procedures, and improving cost-effective management. This section aligns with recent international consensus statements on non-invasive imaging in deep endometriosis.

All tables and figures were carefully reviewed for clarity. The legend of Table 1 was revised to clearly describe the meaning of the numbers presented. The description of Table 2 was corrected to accurately reflect consultation and admission rates per person-year. Figure 3B and Figure 3C legends were adjusted to correctly correspond to the symptoms analyzed, without any modification to the underlying data.

Finally, we expanded the Limitations section to explicitly acknowledge that follow-up was limited to three years after the index surgical event, noting that longer observation periods may reveal different long-term outcomes, recurrence patterns, or shifts in healthcare utilization.

We believe that these revisions have substantially strengthened the manuscript and addressed all reviewer concerns. We are grateful for the insightful feedback and appreciate the opportunity to improve our work.

Corresponding author:

Jose Arnaldo Shiomi da Cruz, MD, PhD

Cincinato Braga St. 37 - Cj 32

01333-011, São Paulo - SP,

Tel/fax : +55 (11) 3294-5592

Email: arnaldoshiomi@yahoo.com.br
